# Reviewer acknowledgement

**DOI:** 10.1186/1475-2875-12-83

**Published:** 2013-04-17

**Authors:** Marcel Hommel

**Affiliations:** 1 Editor-in-Chief, Malaria Journal, Liverpool, UK

## Abstract

**Contributing reviewers:**

*Malaria Journal* would like to thank the following for their assistance with peer-review of manuscripts for the journal in 2012.

## 

Jane Achan

Uganda

Nicole Achee

United States of America

Hans Ackerman

United States of America

Selidji Todagbe Agnandji

Gabon

Bartholomew Akanmori

Congo

Pietro Alano

Italy

Sandra Alba

Switzerland

Audrey Maud Sandrine Albertini

Switzerland

Letusa Albrecht

Brazil

Michael Alifrangis

Denmark

Richard Allen

Australia

Stephen Allen

United Kingdom

Paulo Almeida

Portugal

Evelyn Anane-Sarpong

Ghana

Robin Anders

Australia

Tim Anderson

United States of America

Takeshi Annoura

Netherlands

Nick Anstey

Australia

Paolo Arese

Italy

Gabriela Arévalo Pinzón

Colombia

Elizabeth Ashley

United Kingdom

Peter Atkinson

United States of America

Jo-An Atkinson

Australia

Sarah Auburn

United Kingdom

Lorenz Auer-Hackenberg

Austria

Pinarosa Avato

Italy

Vicky Avery

Australia

Gordon Awandare

Ghana

John Kevin Baird

Indonesia

David Baker

United Kingdom

Kathryn Barbara

United States of America

Bridget Barber

Australia

Karen Barnes

South Africa

Guy Barnish

United Kingdom

Alyssa Barry

Australia

Maria-Gloria Basanez

United Kingdom

Leonardo Basco

France

Christopher Guy Bass

United Kingdom

Quique Bassat

Spain

Imelda Bates

United Kingdom

Jake Baum

Australia

Katja Becker

Germany

James Beeson

Australia

Charlotte Behr

France

Ron Behrens

United Kingdom

John Beier

United States of America

Eric Beitz

Germany

David Bell

Switzerland

Choukri Ben Mamoun

United States of America

Mark Benedict

United States of America

Ramona Benkert

United States of America

Adam Bennett

United States of America

Frances Benson

United Kingdom

Aase Berg

Norway

Elke Bergmann-Leitner

United States of America

James Berkley

Kenya

Klavs Berzins

Sweden

Nora Besansky

United States of America

Ajay Bharti

United States of America

Achuyt Bhattarai

United States of America

Simone Bianco

United States of America

Anne-Lise Bienvenu

France

Beverley-Ann Biggs

Australia

Peter Billingsley

United States of America

Oliver Billker

United Kingdom

Zeno Bisoffi

Italy

Sukla Biswas

India

Anders Bjorkman

Sweden

Simon Blanford

United States of America

Greg Blatch

South Africa

Moses Bockarie

United Kingdom

Steffen Borrmann

Germany

Emmanuel Bottieau

Belgium

Olivier Bouchaud

France

Catherine Bourgouin

France

Teun Bousema

United Kingdom

Zbynek Bozdech

Singapore

Bernard Brabin

United Kingdom

Loretta Brabin

United Kingdom

John Bradley

United Kingdom

Philippe Brasseur

Senegal

Catherine Braun Breton

France

Joel Breman

United States of America

William Brieger

United States of America

Olivier Johan Tavai Briët

Switzerland

Cristiana Brito

Brazil

Rachel Bronzan

United States of America

Basil Brooke

South Africa

Reto Brun

Switzerland

Kwame Ohene Buabeng

Ghana

Pierre Buffet

France

Gerd Burchard

Germany

Tom Burkot

Australia

Alice Butterworth

Australia

Ib Christian Bygbjerg

Denmark

Jens Byskov

Denmark

Adriana Calderaro

Italy

Carlos Campbell

United States of America

Ermanno Candolfi

France

Jorge Cano-Ortega

United Kingdom

Francesco Castelli

Italy

Carlos Eugênio Cavasini

Brazil

Prosper Chaki

Tanzania

Srabasti Jayanti Chakravorty

United Kingdom

Pascalina Chanda-Kapata

Zambia

Daniel Chandramohan

United Kingdom

Sandra Chang

United States of America

Jacques Derek Charlwood

Denmark

Lin Chen

United States of America

Qin Cheng

Australia

Sheleme Chibsa

Ethiopia

Jean-Philippe Chippaux

France

Gerardo Chowell

United States of America

Peter Christensen

Australia

Dennis Christensen

Denmark

Thomas Churcher

United Kingdom

Ian Clark

Australia

Archie Clements

Australia

Lieselotte Cnops

Belgium

Justin Cohen

United States of America

James Colborn

United States of America

William Collins

United States of America

David Conway

United Kingdom

Marc Henri Carl Coosemans

Belgium

Stanton Cope

United States of America

Ross Coppel

Australia

Isabelle Coppens

United States of America

Giampietro Corradin

Switzerland

Patrick Corran

United Kingdom

Vladimir Corredor

Colombia

Michel Cot

France

David Courtin

Benin

Graeme Cowan

United Kingdom

Janet Cox-Singh

United Kingdom

Philip Coyne

United States of America

Alister Craig

United Kingdom

Jakob Peter Cramer

Germany

Ian Crandall

Canada

Pedro Cravo

Brazil

Peter Crompton

United States of America

Juan Cuadros

Spain

Liwang Cui

United States of America

Richard Culleton

Japan

Alexandre Da Silva

United States of America

Valérie D'acremont

Switzerland

Johanna Daily

United States of America

Cyrus Daneshvar

United Kingdom

Jonathan Darbro

Australia

A. P. Dash

India

Claudia Daubenberger

Switzerland

Tim Davis

Australia

Nicholas Day

United Kingdom

Tania De Koning-Ward

Australia

J. Brian De Souza

United Kingdom

Jeremy Debarry

United States of America

Kirk Deitsch

United States of America

Hernando A Del Portillo

Spain

Charles Delacollette

Thailand

Alessandra Della Torre

Italy

Philippe Deloron

France

David Denlinger

United States of America

Evelyn Depoortere

Belgium

Silvia Di Santi

Brazil

Graciela Diap

Spain

Anelia Dietmann

Austria

Carlota Dobano

Spain

Arjen M Dondorp

Thailand

Stefan Dongus

United Kingdom

Martin Donnelly

United Kingdom

Denise Doolan

Australia

Chris Drakeley

Tanzania & UK

Joseph D'silva

United States of America

Jean Pierre Dubost

France

Remy Durand

France

Hillegonda Maria Dutilh Novaes

Brazil

Jeffrey Dvorin

United States of America

Philip Eckhoff

United States of America

Geoffrey Edwards

United Kingdom

Timothy John Egan

South Africa

Stephane Egee

France

Stephan Ehrhardt

United States of America

Daniel Eibach

France

Thomas Eisele

United States of America

Paul Emerson

United States of America

Christian Epp

Germany

Emily Eriksson

Australia

Volker Ermert

Germany

Ananias Escalante

United States of America

Roberto Esposito

Italy

Rick Fairhurst

United States of America

Thierry Fandeur

France

Marit Farenhorst

Netherlands

Jeremy Farrar

Viet Nam

Jean-François Faucher

France

Greg Fegan

Kenya

Ingrid Felger

Switzerland

Heather Ferguson

United Kingdom

David Ferguson

United Kingdom

Marcelo Ferreira

Brazil

Pedro Ferreira

Japan

Marianne Fillet

Belgium

Ulrike Fillinger

United Kingdom

Lawrence Fleckenstein

United States of America

Desmond Foley

United States of America

Adenilson Fonseca

Brazil

Jeffrey Foster

United States of America

Freya Fowkes

Australia

Blandine Franke-Fayard

Netherlands

John Frean

South Africa

Frederic Ariey

France

Michel Frederich

Belgium

Michal Fried

United States of America

Friedrich Frischknecht

Germany

Gabrielle Froberg

Sweden

Mark Fukuda

United States of America

Benoit Gamain

France

F. Javier Gamo

Spain

Don Gardiner

Australia

Simonetta Gatti

Italy

Michelle Gatton

Australia

Deepak Gaur

India

Hellen Gelband

United States of America

Blaise Genton

Switzerland

Annette Gerritsen

South Africa

Peter Gething

United Kingdom

Jose Pedro Gil

Sweden

Philippe Gillet

Belgium

Paul Gilson

Australia

John Gimnig

United States of America

Michael Ginger

United Kingdom

Hagai Ginsburg

Israel

David Gittelman

United States of America

Dean Goldring

South Africa

Iveth Jimena González Jiménez

Switzerland

Catherine Goodman

Kenya

Roly Gosling

United States of America

Marimuthu Govindarajan

India

D. Channe Gowda

United States of America

Patricia Graves

Australia

Michael Green

United States of America

Bryan Greenhouse

United States of America

Brian Greenwood

United Kingdom

Philippe Grellier

France

Jamie Griffin

United Kingdom

Martin Peter Grobusch

Netherlands

Karin Gross

Switzerland

Philippe Guerin

United Kingdom

Lina Andrea Gutierrez Builes

Colombia

Julie Gutman

United States of America

Stefan Hagmann

United States of America

Simon Hales

New Zealand

Rachel Hallett

United Kingdom

Katherine Halliday

United Kingdom

Mary Hamel

United States of America

Davidson Hamer

United States of America

Penelope Hancock

United Kingdom

Thomas Hänscheid

Portugal

Laura Harrington

United States of America

Paul Harrison

Australia

John Harty

United States of America

Emily Harville

United States of America

Ian Hastings

United Kingdom

Susanna Hausmann

Switzerland

Michael Hawkes

Canada

Simon Hay

United Kingdom

Michelle Helinski

United States of America

Olof Hellgren

Sweden

Henri Vial

France

Socrates Herrera

Colombia

Dennis Hesselink

Netherlands

Manuel W Hetzel

Papua New Guinea

Helena Hildenwall

Sweden

Khumbulani Welcome Hlongwana

South Africa

May Ho

Canada

Ary Hoffmann

Australia

Anthony Holder

United Kingdom

Michael Hollingdale

United States of America

Heinrich Hoppe

South Africa

Peter Horby

Viet Nam

Paul Horrocks

United Kingdom

Sandrine Houze

France

Natasha Howard

United Kingdom

Paul Howll

United States of America

Michelle Hsiang

United States of America

Austin Hughes

United States of America

Paul Hunt

United Kingdom

Hilary Hurd

United Kingdom

Lars Hviid

Denmark

Ken Ilett

Australia

Seth Irish

United Kingdom

Jan Jacobs

Belgium

Richard Jähnke

Germany

Keith Jerome

United States of America

Chandy John

United States of America

Jacob Johnson

Kenya

David Jollow

United States of America

Somchai Jongwutiwes

Thailand

Pernille Jorgensen

Laos

Irina Tatiana Jovel

Sweden

Jonathan Juliano

United States of America

S. Patrick Kachur

United States of America

Linda Kalilani-Phiri

Malawi

Seitaro Kamiya

Japan

Rajeev Kaushik

India

Satoru Kawai

Japan

James Kazura

United States of America

Joseph Keating

United States of America

Louise Kelly-Hope

United Kingdom

Richard Kiang

United States of America

Albert Kilian

Spain

Gerry Killeen

Tanzania

Jung-Ryong Kim

Thailand

Jean-Marie Kindermans

France

Kiaran Kirk

Australia

Derryck Klarkowski

Australia

Terry Klein

United States of America

Daniel Kline

United States of America

Robin Kobbe

Germany

Lizette Koekemoer

South Africa

Jacob Koella

United Kingdom

Hannah Koenker

United States of America

Constantianus J.M. Koenraadt

Netherlands

Cristian Koepfli

Australia

Poul-Erik Kofoed

Denmark

Gilbert Onyango Kokwaro

Kenya

Eline Korenromp

Switzerland

Benno Kreuels

Germany

Sanjeev Krishna

United Kingdom

Jürgen Krücken

Germany

Martin Kulldorff

United States of America

Daniel Kyabayinze

Uganda

Sue Kyes

United Kingdom

Dennis Kyle

United States of America

Marcus Lacerda

Brazil

Peter Lackner

Austria

David Lalloo

United Kingdom

Tracey Lamb

United States of America

Abigail Lamikanra

United Kingdom

Jean Langhorne

United Kingdom

Michael Lanzer

Germany

Frederic Lardeux

Bolivia

Miriam Laufer

United States of America

Catherine Lavazec

France

Marc Le Borgne

France

Jean-Yves Le Hesran

United Kingdom

Karin Leder

Australia

Peter Leggat

Australia

Leslie Leiserowitz

Israel

Christian Lengeler

Switzerland

Annick Lenglet

Netherlands

Hoe Nam Leong

Singapore

Toby Leslie

United Kingdom

Chae Seung Lim

Korea South

Kim Lindblade

United States of America

Steve Lindsay

United Kingdom

Bernt Lindtjorn

Norway

Tom Little

United Kingdom

Francesca Little

South Africa

Megan Littrell

United States of America

Neil Lobo

United States of America

Rogelio Lopez-Velez

Spain

Andrew Lover

Singapore

Shirley Luckhart

United States of America

Louise Ludlow

Australia

John Peter Andrea Lusingu

Tanzania

Adrian Luty

France

Sean Lynch

United States of America

Charles Ma

Australia

Musawenkosi Mabaso

South Africa

Vanessa Machault

France

Margaret Mackinnon

Kenya

Amanda Maestre

Colombia

Pascal Magnussen

Denmark

Kathryn Maitland

United Kingdom

Pawan Malhotra

India

Gustavo Malinger

Israel

Jessica Maltha

Belgium

Denis Malvy

France

Sylvie Manguin

France

Ron P. Marchand

Viet Nam

Tanya J Marchant

United Kingdom

Jutta Marfurt

Australia

Kevin Marsh

Kenya

David Marsh

United States of America

Andreas Mårtensson

Sweden

James Mason

United Kingdom

Denise Mattei

France

Barbara Magdalena Matthys

Switzerland

Richard Maude

Thailand

Wilfred Mbacham

Cameroon

Leonard Mboera

Tanzania

Emma Mcbryde

Australia

Matthew Mccall

Netherlands

James Mccarthy

Australia

Margaret Mcconnell

United States of America

John Mcewen

Australia

Rose Mcgready

Thailand

Frank Mckenzie

United States of America

Ushma Mehta

South Africa

Didier Menard

Cambodia

Kamini Mendis

United States of America

Petra Mens

Netherlands

Santiago Merino

Spain

Steven Meshnick

United States of America

Bernard Meunier

France

Sungano Mharakurwa

United States of America

Kristin Michel

United States of America

Nicole Mideo

United States of America

Wilbur Milhous

United States of America

John Miller

Zambia

Paul Milligan

United Kingdom

Noboru Minakawa

Japan

Saroj Mishra

India

Steven Mitchell

Canada

Frank Mockenhaupt

Germany

David Modiano

Italy

Sassy Molylneux

Kenya

Malcolm Edward Molyneux

Malawi

Devanand Moonasar

South Africa

Bruno Moonen

Kenya

Julie Moore

United States of America

Ann Moormann

United States of America

Benjamin Mordmueller

Germany

Luciano Moreira

Brazil

Alexandra Morris

United States of America

Andy Morse

United Kingdom

Eric Mouzin

Switzerland

Godfrey Mubyazi

Tanzania

Atis Muehlenbachs

United States of America

Olaf Mueller

Germany

Ivo Mueller

Papua New Guinea

Ingrid Muller

Germany

Aline Munier

France

Maria Anice Mureb Sallum

Brazil

Sean Murphy

United States of America

Lise Musset

French Guiana

Halima Mwenesi

United States of America

Kesara Na-Bangchang

Thailand

Mathieu Nacher

French Guiana

Bernard Nahlen

United States of America

Denise Naniche

Spain

Wanessa Neiras

Brazil

Ann Marie Nelson

United States of America

Paul Newton

Laos

Raphael N'guessan

United Kingdom

Emanuele Nicastri

Italy

Morten Agertoug Nielsen

Denmark

Harald Noedl

Austria

Gregory Noland

United States of America

Abdisalan M Noor

Kenya

Francine Ntoumi

Congo

Myaing Nyunt

United States of America

Bharti Odhav

South Africa

Bernhards Ogutu

Ghana

Lucy Okell

United Kingdom

Nancy Oleinick

United States of America

Anna Olivieri

Italy

Piero Olliaro

Switzerland

Evelyne Ollivier

France

Wendy O'meara

Kenya

Obinna Onwujekwe

Nigeria

Pamela Orjuela-Sanchez

United States of America

Leonard Ortega

India

Faith Hope Among'in Osier

Kenya

Peter O Ouma

Kenya

Krijn Paaijmans

United States of America

Franco Pagnoni

Switzerland

Sunil Parikh

United States of America

Daniel Paris

Thailand

Gregory Park

United States of America

Robin Parks

Canada

Mercedes Pascual

United States of America

Amy Patterson

United States of America

Rick Paul

France

Christopher Pell

Netherlands

Carlos Penha-Goncalves

Portugal

Freddy Perez

France

Thomas Perry

Canada

Eskild Petersen

Denmark

Margaret Phillips

United States of America

Stephane Picot

France

David Pigott

United Kingdom

Paulo Pimenta

Brazil

Robert Piper

United States of America

John Pisciotta

United States of America

Spencer Polley

United Kingdom

Douglas Postels

United States of America

Bruno Pradines

France

Peter Preiser

Singapore

Ric Price

Australia

Michael Pritsch

Germany

Justin Pulford

Papua New Guinea

Rachel Pullan

United Kingdom

Stuart Alexander Ralph

Australia

Michael Ramharter

Gabon

Milijaona Randrianarivelojosia

Madagascar

Stephane Ranque

France

Hilary Ranson

United Kingdom

Thomas Rask

United States of America

Julian C Rayner

United Kingdom

Husna Razee

Australia

Martin Read

United Kingdom

Michael Reddy

United States of America

Sarah Reece

United Kingdom

Luc Reininger

France

Richard Reithinger

United States of America

Ed Remarque

Netherlands

Laurent Renia

Singapore

Hugh Reyburn

United Kingdom

Arturo Reyes-Sandoval

United Kingdom

Joanna Reynolds

United Kingdom

Jose Ribeiro

United States of America

Jack Richards

Australia

Thomas Richie

United States of America

Robert Ricklefs

United States of America

Natalie L.E. Riedel

Germany

Michelle Riehle

United States of America

Marcus Rijken

Thailand

Eleanor Riley

United Kingdom

Fatima Rivas

United States of America

Vincent Robert

France

Jane Robertson

Australia

Paul Roepe

United States of America

William Rogers

United States of America

Stephen Rogerson

Australia

Christophe Rogier

Madagascar

Petra Rohrbach

Canada

Romain Girod

French Guiana

Philip Rosenthal

United States of America

Christian Roussilhon

France

Alexander Rowe

United States of America

Jose Rubio

Spain

Bruce Russell

Singapore

Tanya Russell

Tanzania

Elizeus Rutebemberwa

Angola

Lora Sabin

United States of America

John Sacci

United States of America

Anavaj Sakuntabhai

France

Ali Salanti

Denmark

Tobili Sam-Yellowe

United States of America

Faruk Sarkinfada

Nigeria

Demba Sarr

United States of America

Robert Sauerwein

Netherlands

David Saunders

Thailand

Allan Schapira

Switzerland

David Schellenberg

United Kingdom

Patricia Schlagenhauf

Switzerland

Wolfgang Hellmut Schmied

Austria

Petra Schneider

United Kingdom

Steve Schofield

Canada

Dirk Schoonbaert

Belgium

Evelin Schwarzer

Italy

Kézia Scopel

Brazil

Anna Seale

United Kingdom

Carlo Severini

Italy

Naman Shah

India

Dennis Shanks

Australia

Y.D. Sharma

India

Surya Kant Sharma

India

Vinod Sharma

India

Todd Shearer

United States of America

Clive Shiff

United States of America

Delane Shingadia

United Kingdom

Addmore Shonhai

South Africa

Carol Sibley

United States of America

Balbir Singh

Malaysia

Neeru Singh

India

Andre Siqueira

Brazil

Jacek Skarbinski

United States of America

Suzanne Skevington

United Kingdom

Tina Skinner-Adams

Australia

Ole Skovmand

France

Larry Slutsker

United States of America

Joseph Smith

United States of America

Thomas Smith

Switzerland

David Smith

United States of America

Georges Snounou

France

Robert William Snow

Kenya

Aurélia Souares

Germany

Tobias Spielmann

Germany

Trine Staalsoe

Denmark

Sarah Staedke

Uganda

Henry Staines

United Kingdom

Danielle Stanisic

Australia

Michelle Stanton

United Kingdom

Rebecca Stanway

Switzerland

Laura Steinhardt

United States of America

Richard Steketee

France

Kasia Stepniewska

Thailand

Mary Stevenson

Canada

Jonathan Stiles

United States of America

Jose Stoute

United States of America

Daniel Strickman

United States of America

Angelika Sturm

Australia

David Sullivan

United States of America

Catherine Sutcliffe

United States of America

Colin Sutherland

United Kingdom

Todd Swarthout

France

Jacek Szepietowski

Poland

Rachida Tahar-Basco

France

Kawsar Talaat

United States of America

Ambrose Talisuna

Kenya

Arthur Talman

United Kingdom

Donatella Taramelli

Italy

Geoff Targett

United Kingdom

Joel Tarning

Thailand

Andrew Tatem

United Kingdom

Wrj Taylor

Thailand

Diane Taylor

United States of America

Terrie Taylor

United States of America

Steve Taylor

United States of America

Andrew Taylor-Robinson

United Kingdom

Fabrizio Tediosi

Switzerland

Annemarie Ter Veen

United Kingdom

Kevin Tetteh

United Kingdom

Sylla Thiam

Kenya

Joanne Thompson

United Kingdom

Anna Thomson

Sweden

Julie Thwing

United States of America

Leann Tilley

Australia

Christian Timmann

Germany

Alfred Tiono

Burkina Faso

Vincent P.K. Titanji

Cameroon

Gabriella Torcia

Italy

Jean Etienne Touze

France

Harold Townson

United Kingdom

Richard Tren

United States of America

Katharine Trenholme

Australia

Marita Troye-Blomberg

Sweden

Takafumi Tsuboi

Japan

Cj Tuijn

Netherlands

V Udhayakumar

United States of America

Rachanee Udomsangpetch

Thailand

Chairat Uthaipibull

Thailand

Glyn Vale

Zimbabwe

Neena Valecha

India

Ana M. Valles-Medina

Mexico

Wim Van Bortel

Belgium

Katarzyna Van Damme-Ostapowicz

Poland

Anna Maria Van Eijk

Ethiopia

Jean-Pierre Van Geertruyden

Belgium

Ann Van Schepdael

Belgium

Jerome Vanderberg

United States of America

Veerle Vanlerberghe

Belgium

Ashley Vaughan

United States of America

Jefferson Vaughan

United States of America

Meera Venkatesan

United States of America

Jonathan Vennerstrom

United States of America

Hans Verhoef

Netherlands

Federica Verra

United Kingdom

Joseph Vinetz

United States of America

Lorenz Von Seidlein

Australia

John Vontas

Greece

Francis Wafula

Kenya

Mats Wahlgren

Sweden

Patrick Walker

United Kingdom

Edward Walker

United States of America

Catherine Walton

United Kingdom

Ping Wang

United States of America

David Warhurst

United Kingdom

Norman Waters

United States of America

Marcia R Weaver

United States of America

Jayne Webster

United Kingdom

Walter Weiss

United States of America

Timothy Wells

Switzerland

David Wenkert

United States of America

Walther Wernsdorfer

Austria

Bradley White

United States of America

Michael White

United Kingdom

Nicole Whitehurst

United States of America

Christopher Whitty

United Kingdom

Kim Williamson

United States of America

Mark Wilson

United States of America

Michael Wilson

United States of America

Ernst A. Wimmer

Germany

Tonia Woodberry

Australia

Charles Woodrow

Thailand

Colin Wright

United Kingdom

Gerhard Wunderlich

Brazil

Michelle Wykes

Australia

Rajpal Singh Yadav

Switzerland

Anjali Yadava

United States of America

Stephanie Yanow

Canada

Shunmay Yeung

United Kingdom

Sedigheh Zakeri

Iran

Fidel Zavala

United States of America

Herve Zeller

Sweden

Guofa Zhou

United States of America

Peter Zimmerman

United States of America

Thomas Zoller

Germany

Dejan Zurovac

Kenya

Julien Zwang

Switzerland

